# Unique models of embodied cognition and eco-social niches proposed to validate hypothesis of social attunement and mis-attunement with a focus on autism

**DOI:** 10.3389/fpsyt.2025.1562061

**Published:** 2025-05-23

**Authors:** Amit Kumar

**Affiliations:** Strategic Technology Services, Technology Transformation, Infosys Consulting, Infosys (United States), Palo Alto, CA, United States

**Keywords:** autism spectrum disorder, embodied cognition, action perception, action control, eco-social niche, hidden state, attunement, mis-attunement

## Abstract

This paper uses basic concepts of embodied cognition based on the idea that the biology of the brain is impacted by the physics of movement and the interacting physical objects as well as biological markets or competition. These concepts are leveraged to explain the emergence of autism features and characteristics through multiple hypotheses using hidden states and eco-social niche. I begin by defining cognitive granularity, which forms the basis of my embodied cognition hypotheses. These hypotheses leverage cognitive psychology concepts to distinguish three dyads—resource proxy, executor, and evaluator—each with different sensorimotor skills for action control and action perception. Each of these dyads interacts with the environment in physically distinct and beneficial ways leading to iterative honing of the dyads’ individual skills: dialects of internalization and externalization. Collectively, these interacting dyads further form the basis of social attunement and mis-attunement—dialects of individual and collective. The social attunement hypotheses apply economic concepts of supply/demand curve and Nash equilibrium or game theory to the evolving human (hominin) biological market to derive eco-social niches for modeling the underlying neurotypical dyads: executor and evaluator. I hypothesize that insufficient sensorimotor integration within a dyad impedes proper participation in the eco-social niche, leading to psychiatric issues. In my model, this mis-attunement is reflected in the characteristics of the resource proxy and extreme evaluator dyads.

## 1 Introduction

Although extensive genetic, clinical, and theoretical research (1) has advanced our understanding of autism, its true causes remain elusive. This paper integrates current autism research with new hypotheses, paving the way for further study. The hypotheses proposed in the paper are based on case studies, empirical observations, and the synthesis of available autism and cognition literature. This paper elaborates further on the latest thinking on autism, which is beyond the boundaries of the individual mind:

Multi-scale, fine-grained analysis of social interaction might help us elucidate the underlying behavioral and neural mechanisms both at levels of individual, but also at the level of interacting bodies and minds. [Boli, Dumas, Schillbach, 2022, 1]

The paper begins by summarizing the key theories of autism while providing an overview of the proposed hypotheses as they relate to new areas of autism research, in particular, the multi-dimensional space of the individual and collective as well as models of attunement and mis-attunement (2). Attunement is when a pair of dyads are confirming to an eco-social niche and mis attunement occurs when a dyad is not in resonance with the set of co-evolving dyads that form the niche. There are three parts to the hypotheses—one group of hypotheses focuses on the variations in the hidden states of embodied cognition across neurotypical hominin and neurodivergent dyads to model the internal neural dynamics^2^, or information processing capabilities; the second group focuses on the models of eco-social niche to model the external forces; and the third focuses on attunement and mis-attunement among these models, which are outlined in further detail below.

- Group 1: Hypotheses leverage the cognitive psychology concepts of embodied cognition, active inference, sensorimotor skills of action perception, and action control using motion or movement as the unifying factor to build generative hidden state hypotheses. My hypotheses build on documented sensorimotor differences in action perception and control. I use these differences to construct a spectrum of hidden states across Hominin male individual, Hominin female individual, and autism spectrum disorder (ASD) populations. Another important hypothesis is related to the correlation of variation of motion sickness across the hidden state spectrum defined above and is of great significance because of the association of psychiatric illness with susceptibility to motion sickness (3).
- Group 2: Biological markets reflect economic principles observed in mating behaviors (4). Hominin males individuals cooperation and competition can be modeled using a supply curve, while homin-female individuals selection process corresponds to a demand curve model to “choose” hominin males (“chosen”) to reap the best survival outcomes (4, 5).
- Group 3: How does neurodivergence (psychosis and autism) fit into all this? In neurodivergent populations (such as ASD), key features required to align with these eco-social niches are absent. For example, individuals may show deficits in the theory of mind, executive function^3^, and sensorimotor integration. These issues manifest as an inability to “match” the economic model’s optimal behavior, leading to mis-attunement. An extreme form of “choosing”, characterized as an extreme “evaluator”, is hypothesized to parallel psychosis. Therefore, the Group 3 hypothesis basically articulates the mis-attunement of the features that make up the eco-social models as well as the extreme spectrums of the embodied cognition hypotheses.

These hypotheses can serve as inputs to the multi-scale dimensional model hypothesis of social attunement and mis-attunement and thus serve the end of interpersonal psychiatry as well as a holistic model or theory to explain the evolution and heterogeneous features of autism. Additionally, my reference to “hominin male” and “hominin female” as “executor” and “evaluator” dyads, respectively, throughout the paper should be treated as referring to evolving humans over millennia, which had more segregated roles but evolved into having more homogenous and beneficial traits agnostic of the sex in the present time but with some lingering vestigial traits that can offer a clue into how we evolved and more importantly the social evolutionary processes that led to the evolution of ASD. Even the reference to “resource proxy” is purely theoretical to draw a correlation to the atypical object cognition characteristics of ASD and in no way intended as insensitive treatment of the individuals with ASD.

## 2 Current research developments and existing theories of autism spectrum disorder

Current autism research is heavily focused on identifying the genetic components of ASD, with studies finding a significant role of genetic variations (1, 6) in a large percentage of cases, and is also exploring the potential impact of environmental factors (7, 8) on specific autism traits; researchers are also investigating brain differences (9, 10) in autistic individuals, including variations in cortical thickness and synapse density, which could lead to better understanding of the neurological basis of ASD; additionally, research is highlighting the need for diverse genetic databases to accurately interpret data across different populations (11). In addition to genetic and brain imaging research, there is a significant focus on ASD as a multi-person mis-attunement issue. The crucial new development in psychiatry and, in particular, autism is the move from single-person diagnosis to collective attunement and mis-attunement. Therefore, there is a paradigm shift in how psychiatric issues will be evaluated in the future, i.e., interpersonal psychiatry (2), and this paper proposes testable hypotheses to support this new development in the field of psychiatry, especially with the advent and progress in artificial intelligence and machine learning^4^.

Multiple theories of autism have been proposed over the years, which individually and collectively do explain some ASD features, and attempts have also been made to formulate a unified theory (12–14), but a more inclusive and testable theory that explains the evolution as well as clinically reported heterogeneous features is still not formulated. Additionally, some of these theories contradict each other and do not offer consistent experimental evidence.

### 2.1 Extreme male theory

Autism has historically been diagnosed four times more often in men than in women. There are also certain cognitive domains that vary by sex: women tend to excel at empathizing, and men tend to excel at systemizing. These two principles form the foundation of psychologist Simon Baron-Cohen’s theory (15, 16) that autism represents an “extreme” form of the male brain.

### 2.2 Imprinted brain

Imprinted genes refer to genes expressed from one parent rather than from both parents. According to the imprinted brain theory (17), paternal gene expression may cause a child to have a larger brain, develop more quickly, and demand more from the mother. Maternal gene expression may cause a baby to have a smaller brain, develop more slowly, and demand less from the mother.

The extreme male brain theory stands in opposition to the imprinted brain theory, which stipulates that autism is the result of more paternal gene expression rather than exaggerated male brain development. Some research has failed to find anatomical evidence (18) that men with autism have extreme male brain development. One is that sex differences may not exist or be as substantial as needed to support the theory—they may be very small. Assessment tools may also center on topics that boys are socialized to use, such as machines.

### 2.3 Social motivation hypothesis

The social motivation hypothesis (19) proposes that autism may be due to differences in the brain’s reward system. Neurotypical individuals find social interactions, such as eye contact and conversation, inherently valuable. Autistic individuals find social interactions difficult or uninteresting. Some research supports this hypothesis: children with autism show less reward-related brain activity when anticipating social information than neurotypical children do.

### 2.4 Weak central coherence

This theory suggests that people with ASD have difficulty seeing the big picture and focus on parts instead of the whole. The weak central coherence theory (20) of autism attempts to account for both deficits and strengths in perceptual processes and refers to the phenomenon that diagnosed individuals show a bias for featural and local information, failing to extract the gist or “big picture”. This detail-focused cognitive style leads to potential superiority in local or detail-focused processing. Performance on visuospatial, perceptual, and verbal-semantic tasks, which require strong central coherence, is impaired in ASDs that support this theory (Ropar and Mitchell, 1999; Happe and Frith, 2006).

### 2.5 Executive dysfunction theory

Executive functions include planning and organizing, initiating behavior or activity, switching focus, self-regulation, and impulse control (Boucher, 2009; Cumine et al, 2009). This theory suggests that in people with autism, there is a degree of dysfunction in these processes, and this results in some of the difficulties people on the spectrum experience with motivation, coping with change, self-regulation, and control as well as an impact on practical daily life skills that rely on good self-organization and planning such as dressing, shopping, and cooking.

### 2.6 Intense world theory

The intense world theory (14) is that people with autism have increased brain activity, which makes it hard to selectively pay attention to certain things and not others. They may experience the world as more intense or overwhelming than neurotypical people. For example, at a party, it may be difficult for neurotypical individuals to focus on the one person they are speaking to and ignore everyone else. For someone with autism, the sound of an air-conditioner could feel grating, or a sweater could feel itchy. This idea would mean that two core features of autism—social challenges and sensory sensitivity—may both be rooted in overactive brain responses.

### 2.7 Hyper-systemizing theory

The hyper-systemizing theory (21) posits that people with autism are hyper-systemizers: individuals who are motivated to identify lawful regularities that govern the input-operation-output workings of a system. Hyper-systemizing is posited to potentially contribute to savant skills in the domains of calendrical calculation, mathematics, and music due to obsessive systemizing in that domain.

### 2.8 Diametric mind theory

This theory (22) holds that autism and psychosis exist on opposite ends of a spectrum. Autism leads to an understanding of the world in literal, mechanistic terms, and a limited ability to understand others’ intentions and perspectives. Psychosis, in contrast, results in the “overinterpretation” of other’s intentions, misreading minds to such a degree that paranoia and delusion result.

Brain imaging demonstrates that autism and psychosis have opposite influences on brain activity in regions that govern mentalizing and reorienting attention. Autism is associated with decreased activity in those areas, and psychosis is associated with increased activity in those areas.

### 2.9 Theory of mind

The “theory of mind” (ToM) model (23) suggests that people with ASD have profound difficulty understanding the minds of other people—their emotions, feelings, beliefs, and thoughts. As an explanation for some of the characteristic social and communication behaviors of people with ASD, this model has had a significant influence on research and practice. This deficit is often called “mind blindness”. The “false-belief task” is often used to probe children’s developing theory of mind. After the first test of this ability in young children by Wimmer and Perner (1983), numerous versions of the false-belief task have been used with preschool-aged children.

Most of these theories have honed into some of the symptoms of autism and proposed a theory to model the symptoms. Except for the theory of mind, all theories are focused on autism as an individual or single-person problem, but recently, there has been a change in the research outlook by framing ASD as a multi-person attunement or mis-attunement issue, and this paper proposes unique models of embodied cognition and eco-social niches to outline a broad-based and unified theory of autism.

## 3 Unique models of embodied cognition and eco-social niches leading to attunement and mis-attunement

**Figure 1:**
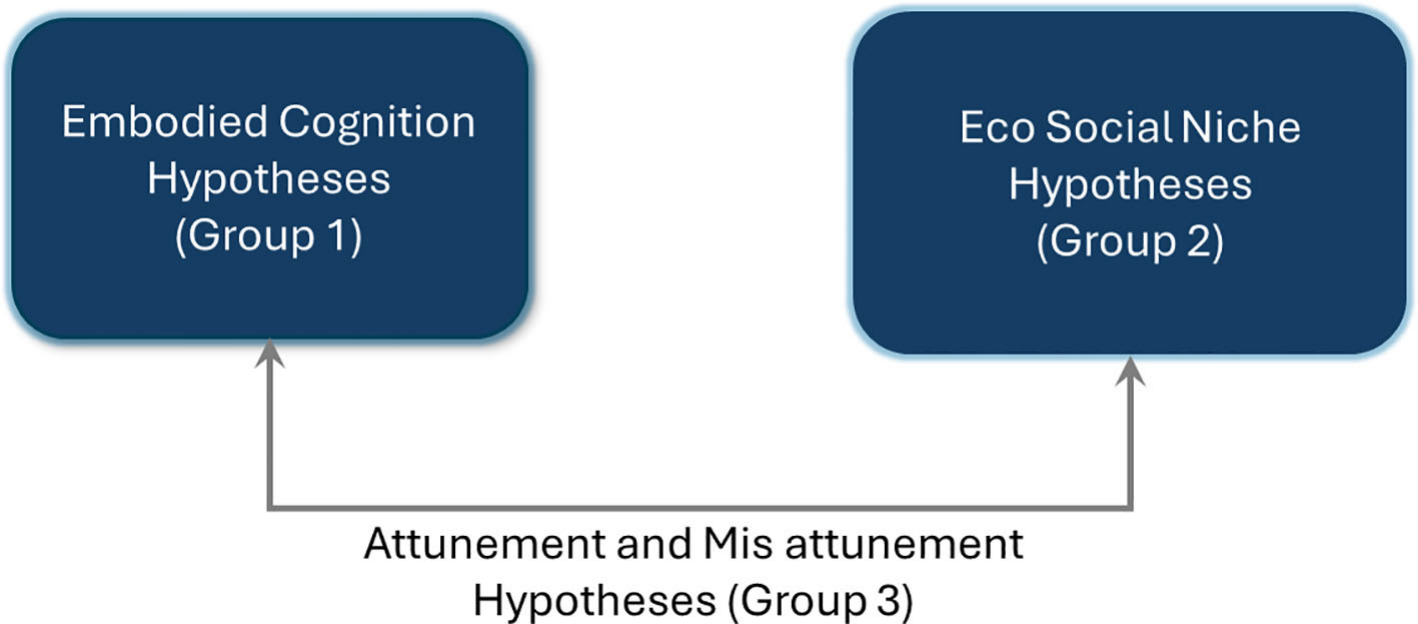
Co-evolution of embodied cognition and eco-social niches - 3 groups of hypotheses.

As we progress toward this new thinking and proposal of embodied cognition as well as eco-social models, it is important to acknowledge that the above theories are of immense importance and do provide the foundation for the next generation of theories and models. The approach proposed in this paper is unique, as it not only outlines hypotheses for internal embodied cognition (Group 1) but also external eco-social niches (Group 2) that interact with each other in specific ways such that they can be modeled and serve as proxies for attunement or mis-attunement (Group 3) as shown in **Figure 1**. The above autism theories *of intense world, hyper-systemizing, diametric mind, executive dysfunction, and theory of mind* are part of these hypotheses but are connected in a unique way such that they can be tested using artificial intelligence/machine learning (AI/ML) models (2).

The dialectics of internalization as well as externalization (2) explore how they are interconnected and essential for understanding the self and social reality. This paper defines three hidden internal states (24)—evaluator, executor, and resource proxy, or the internal neural dynamics that exert varying levels of sensorimotor control, i.e., action perception and action control (25, 26), on the environment. My paper first establishes five hypotheses in Group 1, namely, hypotheses 1, 2, 3, 4, and 5, on the psychophysiological^5^ internal structure, i.e., how the uniqueness of internal states in turn uniquely impacts the external environment or external objects. These dyads use basic physics concepts and continue to further chisel the internal states through dialectal interaction to reduce prediction errors through fine-tuned sensorimotor control (27). The concept of “the dialects of the individual and the collective” further discusses:

The above-mentioned hierarchical structures of predictive processing should be considered as collectively shaped. First, we dynamically ‘embody’ each other in and through social interaction, enabling interpersonal attunement (e.g. interpersonal belief resonance). In other words, by engaging in sensory-motor couplings with others in social interactions, we have our bodily structures mutually transformed beyond the here and now. Second, such structures, arguably, unfold within nested time and space scales, from biology and cognition all the way up to society. Taken together, humans actively co-construct and co-regulate—in interaction with other organisms—their Eco social niches, so that they increase survival chances of not just the individual, but also the social group and the species as a whole. [Boli, Dumas, Schillbach, 2022, 3]

Additionally, I draw on the above concept in this article to outline my hypotheses related to the collective interaction of the above three hidden states (28)—resource proxy, executor, and evaluator—each of which has a population of multiple dyads interacting in specific ways among themselves and with the dyads from other hidden states. My paper establishes a co-opetition eco-social niche hypothesis, namely, hypotheses 6 and 7, among these dyads by leveraging the economic supply and demand curve (29) and Nash equilibrium (29, 30) to establish Group 2. Finally, Group 3 includes the attunement as well as the mis-attunement hypotheses, first introducing hypothesis 8, which, together with hypotheses 2, 3, 4, 5, 6, and 7, forms the interpersonal attunement framework that functions in cognizance to establish a multi-dimensional eco-social niche. The concept of “interpersonal mis attunement in and through social interaction” (2) further discusses:

So far, the paper has considered the importance of interpersonal attunement in social interactions and the formation of the human self. Subsequently, placing the focus on psychopathology, the paper extends the discussion from attunement to potential mis attunement, discussing the dialectical mis attunement hypothesis. According to this hypothesis, psychopathology can be viewed not as mere (mis-) function within single brains, but also as a dynamic interpersonal mismatch (for a comprehensive review of the phenomenon as well as the relevant psychophysiological processes). The primary aim of such an approach is to move beyond the individual in the study of psychopathology, yet without neglecting the tightly connected psychophysiological processes at play. Of note, an interpersonal mis attunement, as defined above, lies in the interaction between the two parties and as such it constitutes a collective phenomenon non-reducible to either of them. Here, it is important to underline that this kind of interpersonal mis attunement should be treated as a phenomenon at the intersection of the individual and the collective. [Boli, Dumas, Schillbach, 2022, 4–5]

To continue to draw from this mis-attunement concept, I have outlined my final two Group 3 hypotheses (hypotheses 9 and 10), which outline the special characteristics of the resource proxy dyads and extreme evaluator dyads that cause mis-attunement of the eco-social niche outlined previously.

## 4 Applicability of market economic theories of demand/supply and Nash equilibrium to biological markets

The key elements of economics include scarcity, supply and demand, opportunity cost, costs and benefits, incentives, and market dynamics, essentially focusing on how individuals and societies make choices regarding the allocation of limited resources due to the concept of scarcity, which is a fundamental principle in economics. While traditional economic theory cannot be directly applied to biological markets in its entirety (31), many concepts from economics, particularly those related to game theory and exchange, can be used to understand and model interactions between organisms in ecological systems, especially when considering mutualistic relationships where organisms exchange resources, often referred to as “biological markets” (31); however, key differences like the lack of enforceable contracts and complex biological constraints need to be considered when applying these concepts. Both economic and biological markets involve individuals (organisms) making choices to exchange goods (resources) with the goal of maximizing their own benefit, often leading to competition and cooperation dynamics. Some examples of the applicability of economic concepts to biological markets are as follows:

- Mating markets: studying mate selection in animals using concepts like “supply and demand” to analyze how individuals choose partners based on their traits and availability.
- Mutualistic symbiosis: examining interactions between species where both organisms benefit from resource exchange, such as mycorrhizal fungi providing nutrients to plants in exchange for carbohydrates, using models similar to “barter trade”).
- Principal–agent problem: analyzing situations where one organism (the “principal”) relies on another (the “agent”) to perform a service but needs to ensure the agent acts in their best interest, which can be seen in certain host–parasite relationships.

**Figure 2:**
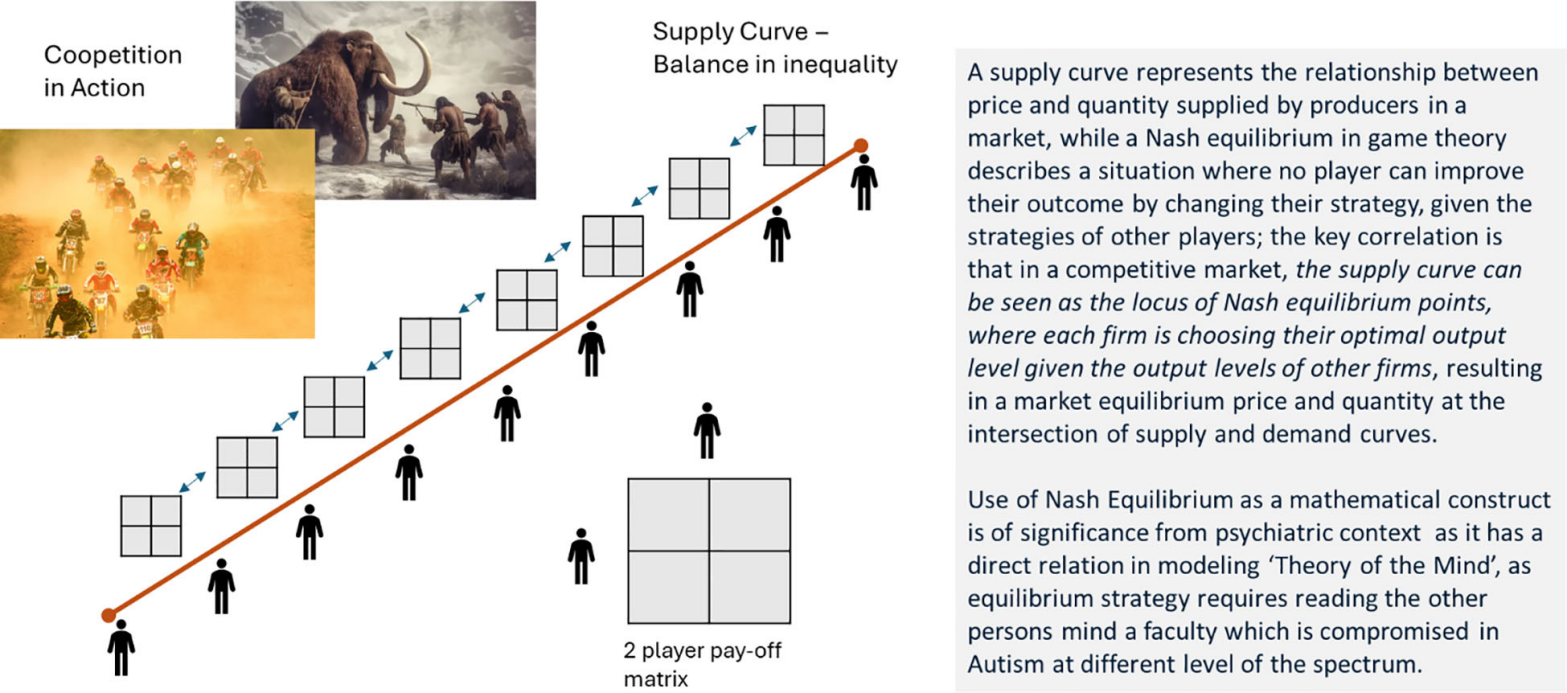
Supply curve and significance of Nash equilibrium in the psychiatric context.

This paper leverages areas where economic concepts are applicable to biological markets, especially the *mating markets* and concepts of supply/demand and game theory—Nash equilibrium. A supply curve represents the relationship between price and quantity supplied by producers in a market, while a Nash equilibrium in game theory describes a situation where no player can improve their outcome by changing their strategy, given the strategies of other players. The key correlation is that in a competitive market, the supply curve can be seen as the locus of Nash equilibrium points, where each firm chooses its optimal output level given the output levels of other firms, resulting in a market equilibrium price and quantity at the intersection of supply and demand curves (32). To model human interaction while still upholding the principles of economic theory, I will leverage biological market concepts, as defined in the role of supply and demand in biological mating markets (33), but I am proposing the substitution of producers and firms in the above definition with hominin males to model the biological supply curve, as shown in **Figure 2**.

In that context, the inclusion of hominin males (and not hominin females) is a safe assumption from a biological market modeling standpoint of one of the markets—an arms race of males in cooperation and competition (34) or cooperation markets: males trading with conspecific males (4). Contemporary social underpinning based on modern culture and inclusivity has removed the distinction between the evolutionary division of labor between male and female, but for the longest period in human history, the roles were pretty differentiated between male and female of the species—male as the chosen and female as the chooser (4, 5). Further evidence for Nash equilibria arises from studies that have shown that Nash equilibria arise naturally in motor interaction in which players vie for control and try to minimize effort—this was demonstrated through the rope pulling and classical prisoner’s dilemma task (35).

## 5 Cognitive granularity is defined as a variation in sensorimotor skills (action control and action perception) and is impacted by biological motion

**Figure 3:**
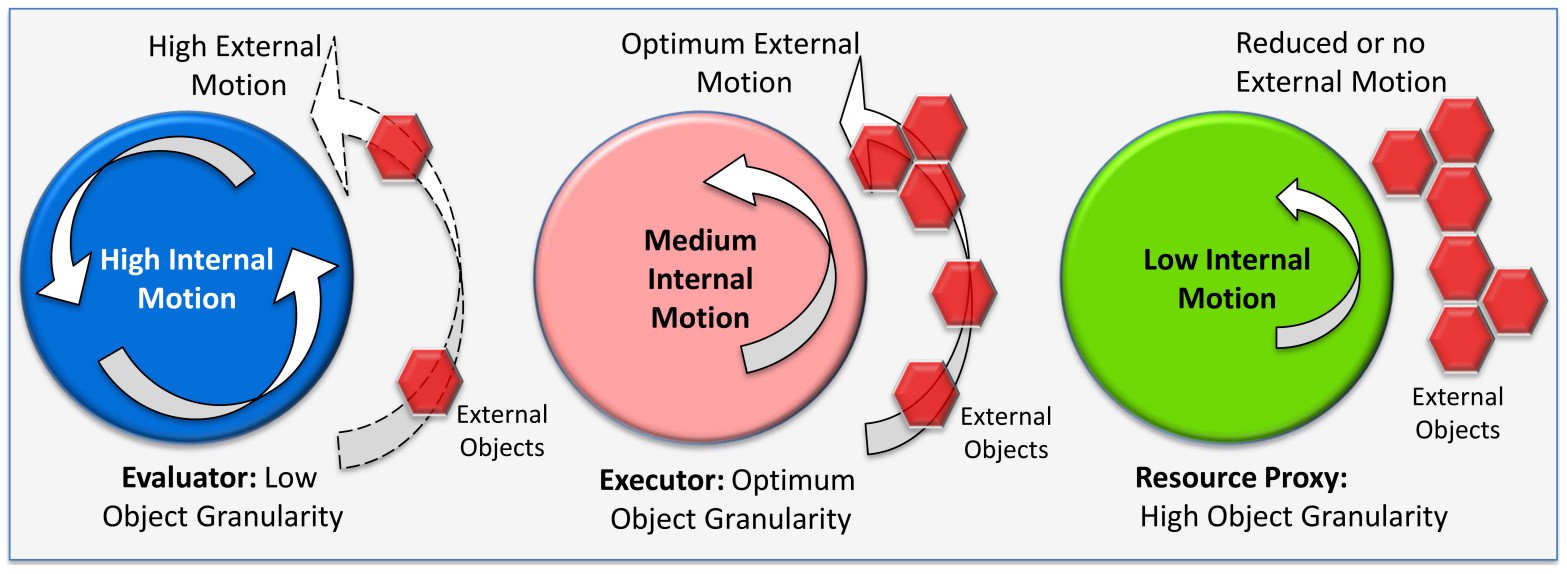
Spectrum of hidden state model of embodied cognition.

I introduce a new term called cognitive granularity, which defines the level of external object affiliation of a person governed by biological motion or movement. An autistic person generally exhibits superior systemizing skills (16) with respect to external objects or images and, per my proposed terminology, would be a person with high cognitive granularity. I map neurodivergent and neurotypical on a cognitive granularity scale, as shown in **Figure 3**, with variation in movement as a unifying factor across the spectrum. Therefore, a neurodivergent-autistic individual would be high on the cognitive granularity scale, a neurodivergent psychotic would be low on the cognitive granularity scale, and a neurotypical would have an optimum level of cognitive granularity. I leverage the concept of embodied cognition (36), which has roots in motor control and emphasizes that cognition typically involves acting with a physical brain (shown as circles in **Figure 3**) on the environment (shown as red hexagonal objects in **Figure 3**) in which the body is immersed leveraging a physics concept of gear belt pulley^6^ (37). Circles in **Figure 3** are equivalent to the motorized wheel, and the hexagon objects in the environment are equivalent to the other wheel connected through our limbs (not shown in the schematic) to transfer a proportional level of energy to the environment (38). The schematic in **Figure 3** is a conceptual diagram or model to represent the varying hidden states for developing the hypotheses. *This also helps develop an intuition of how nature* sp*ecialized the neural dynamics or information processing capabilities for variation in sensorimotor skills or eco-social niches across the dyads*. Research is underway to uncover the internal model that predicts the physics of an object by studying the brain’s neural network and p-cells in the cerebellum and the motor cortex, but the alien language used by the brain to predict and control the physics of our body is still not deciphered (24). Experiments have been conducted using an “error-clamp” (24) to allow exquisite quantification of the process of learning physics—the coordinate system of within-arm generalizations appeared to depend largely on the neural representation of proprioception with *strong sensitivity to velocity* but poor encoding of acceleration (24). The cognitive model in **Figure 3**, developed heuristically based on available information in literature, leverages the variation in internal motion or velocity to propose multiple specialized dyads that have a varying impact on the objects in the environment. All this is important, as the orientation of the p-cell and overall motor function is compromised in ASD, so an internal cognitive model hypothesis based on internal motion variations can help translate phase shifts in the neural network dynamics between neurotypical hominin and ASD.

## 6 Mental hidden state hypotheses that impact the external environment in specific ways

**Figure 4:**
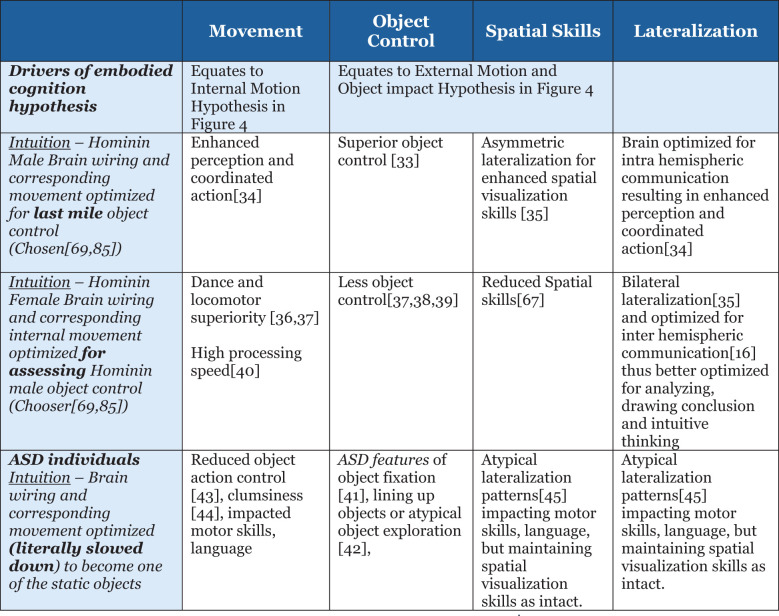
Summary of variation in current brain or information processing offering a clue to hominin evolution.

Uncovering hidden brain states and their dynamic spatio-temporal evolution in relation to cognitive task demands, i.e., information processing, remains an important unresolved problem in human cognitive neuroscience research (39). The hidden state hypotheses proposed in this paper are based on facts available in the literature as well as multiple studies (40–47) on gender differences in processing speed, object control, spatial skills, and sensorimotor skills: for *males*, superior object control (40), brain optimized for intra-hemispheric communication resulting in enhanced perception and coordinated action (41), and asymmetric lateralization for enhanced spatial visualization skills (42); for *females*, dance and locomotor superiority (43, 44) but less object control (44–46), reduced spatial skills (48), and bilateral lateralization (42) and optimized for inter-hemispheric communication (11) and thus better optimized for analyzing, drawing conclusion and intuitive thinking, and high processing speed (47); and for *ASD features* of object fixation (49), lining up objects or atypical object exploration (50), reduced object action control (51), clumsiness (52), and atypical lateralization patterns (53) impacting motor skills and language but maintaining spatial visualization skills as intact—further summarized in **Figure 4**.

Studies (54) of the visual perception of biological motion have, thus, been thought to provide a window into social dysfunction in ASD. With regard to the visual processing of others’ actions, disruptions in the visual perception of biological motion have been discussed as a potential hallmark of ASD (55). Here, an impaired sensitivity for processing the actions of others (as compared to observing objects) has been demonstrated (56). These findings were also paralleled by neuroimaging results indicative of atypical neural response patterns to biological motion perception with point-light stimuli (57). Such point-light displays are created by attaching markers to a person’s body and head and then recording that person’s movements so that only the point lights are visible.

Considering these information processing data points from the literature, I have consolidated all these characteristics in a simplified but insightful pattern of cognitive task demands and hidden states, as shown in **Figure 3**. I was motivated by the idea that the biology of the brain is impacted by the physics of moving and interacting physical objects (24), so there is a direct connection or energy transfer (38) between internal motion and external motion. This gives us a peek into the variability of the mind for a thorough analysis and validation through AI/ML algorithms and mathematical models (2, 58). I have hypothesized that the hidden states (28) of neural dynamics range from high to low motor control. I am quantifying motor control as high internal motion, optimum internal motion, and low internal motion. Each of these has an equivalent and directly proportional external motion coupling (like a gear belt pulley for transferring motion and power (38)). In effect, we are extrapolating physics principles (24) into the brain to act in specific and predictable ways on the object in the environment. However, extremes of these internal motions result in neurodivergent behavior.

The paper also proposes a connection between the proposed cognitive internal models or hidden states (**Figure 3**) and susceptibility to motion sickness. The primary theory of motion sickness proposes that motion sickness is a mismatch between sensory information received from different parts of the body, such as the inner ear (vestibular system), which detects movement, while the eyes see a stationary scene, creating a conflicting signal to the brain (59–61). Embodied cognition proposes that our brain constantly builds an “internal model” of our body’s movement based on sensory feedback, and when this model is disrupted by conflicting information, it can trigger motion sickness symptoms. No clear internal model (62) has been proposed that explains the occurrence of motion sickness and its variability among the genders. The model proposed in **Figure 3** can be used as a basis for explaining the variations in motion sickness in relation to the variation in internal motion. We know that females in general have a higher susceptibility (63) to motion sickness and that motion sickness susceptibility can be used as a neurophysiologic index to establish a polar spectrum of personality differences (3). The latter point is of great significance before I outline the embodied cognition hypotheses, as studies (3) have shown that susceptibility to psychiatric illness (schizophrenia and psychosis) correlates with motion sickness susceptibility and aligns with the mis-attunement hypothesis 10. Based on the evaluation of 2,032 psychiatric inpatients, the paper (3) concludes:

There appears to be little question that some kind of CNS (*Central Nervous system*) integrative vulnerability fosters the development of psychotic illness in the young persons found in the mid-spectrum of motion sickness susceptibility. This illness is more common in males and essentially diagnosed clinically as schizophrenia. It is apparent that persons at the extremes of motion sickness susceptibility, by virtue of the contrasting but more stable organization of their CNS, are protected against this form of illness but are vulnerable to a different kind of disorder somewhat later in life. This illness is more common in women and is often diagnosed as an affective psychosis. [Charles M., Bernard G., 1980, 46]

The authors of the paper (3) further conclude:

A substantial amount of evidence suggests that persons who are not susceptible to motion sickness are more embedded in and responsive to their environments, while susceptible persons are more autonomous and self-contained. [Charles M., Bernard G., 1980, 45–46]

This experimental evidence of the susceptibility to motion sickness aligns with the object-agnostic features of extreme evaluator and executor features of being tuned into the external objects. Motion sickness susceptibility in the context of the embodied cognition model (**Figure 3**) is important, as it helps to establish hypotheses that can be tested by the magnitude of motion sickness symptoms as I traverse this spectrum in **Figure 3**.

### 6.1 Group 1: Embodied cognition hypotheses

#### 6.1.1 Hypothesis 1: ASD hidden state hypothesis for action control

The resource proxy dyad (neurodivergent autistic or high cognitive granularity) is represented by the green (rightmost) circle in **Figure 3**. It is hypothesized to exhibit “low internal motion” due to reduced motor control (53). This reduced internal motion leads to lower external motion, causing “stickiness” in external objects (49) and ultimately reduced action control (51). Therefore, the resource proxy dyad, as the name suggests, can be intuitively and characteristically envisioned as “one of the static objects” in the environment; thus, it is not acting on the actual objects optimally, as it lacks the motion needed for strong coupling.

#### 6.1.2 Hypothesis 2: Hominin female hidden state hypothesis for action control

Evaluator-hominin female or low cognitive granularity dyad is represented by a blue circle (leftmost) in **Figure 3**. It is hypothesized to have a “high internal motion” hidden state of neural dynamics because of increased processing speed (47). This leads to a specific impact on the external objects represented as hexagons in the environment, namely, causing the object to “run or slip”, resulting in reduced (45, 46) object action control due to “high external motion”. This is represented by multiple hexagon objects sparsely spread out or rarified. Therefore, the evaluator dyad can be intuitively or characteristically envisioned as “agnostic of the object or removed from the object” or has limited connection with the objects and reduced spatial visualization skills (64).

#### 6.1.3 Hypothesis 3: Hominin male hidden state hypothesis for action control

The executor or optimum cognitive granularity dyad is represented by a pink circle (middle) in **Figure 3**. It is hypothesized to have “optimum internal motion” (40) neural dynamics or motor skills (41). This results in “optimized external motion” or optimum action control (65) on the red hexagon objects in the environment, as if these objects are an extension of their own bodies (66, 67) during physical execution. Therefore, the executor dyad can intuitively be envisioned as “one with the moving object in motion” or optimum object coupling to deftly exert action on the object(s). The use of the term “optimum” neural dynamics refers to nature’s natural selection process that yields sustained beneficial survival outcomes.

#### 6.1.4 Hypothesis 4: Action perception hypothesis

Due to the varying neural dynamics of the brain in the three dyads, action perception is also variably encoded across the above spectrum to enable the variation in the action control. Action perception is directly proportional to the hidden state of “internal motion”, so the evaluator dyad will have superior action perception (45) at the cost of reduced action control (45, 46), the resource proxy dyad will have reduced action perception (54) as well, and the executor dyad will have optimum action perception (41, 50, 65).

#### 6.1.5 Hypothesis 5: Motion sickness hypothesis

Due to the varying neural dynamics of the brain in the three dyads, motion sickness is also related to the cognitive internal model or hidden state. Motion sickness is directly proportional to the hidden state of “internal motion”, so the evaluator dyad has a greater susceptibility to motion sickness due to the additive impact of external motion to an already high internal motion hidden state, the resource proxy dyad will have reduced motion sickness, and the executor dyad will have limited motion sickness.

**Figure 5:**
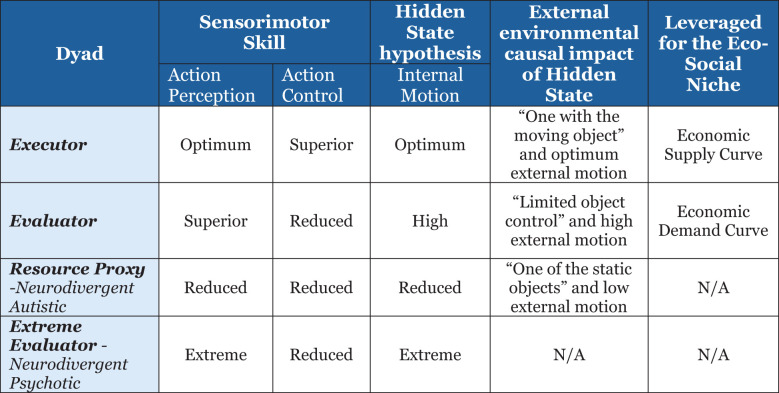
Correlation of sensorimotor skills with hidden states and impact on the environment (feature space).

From active inference (25, 26), we know that action control and action perception work in concert to minimize a shared functional known as variational free energy^7^. Therefore, based on the above hypotheses, cognitive granularity is impacted by the variation in the sensorimotor skills of action perception and action control, as summarized in **Figure 5**. As a result, the executive function of an individual on this spectrum is compromised (executive dysfunction theory) when cognitive granularity is on an extremely high or extremely low scale, but the executive function is normal when the cognitive granularity is just optimum.

## 7 Co-evolution of the individual mental hidden states and the eco-social niche hypotheses

These individual sensorimotor traits of action perception and action control in humans have developed in response to external environmental conditions and evolutionary demands (27). These external conditions may seem too extensive and numerous to model; therefore, the additional hypotheses (hypotheses 6,7, and 8) focus on three evolutionary external needs or demands that may have impacted or influenced the evolution of sensorimotor skills in the specific ways defined in the previous section. Many theoretical papers discuss the interaction of the individual and the shaping of the collective interactions (2, 40, 68) in the formation of societies (69). Hypotheses 6 and 7 discriminate this further by identifying a range of fine-tuned societal specializations motivated by external demands. One is co-opetition-based collective execution (70) societal specialization formed by a collection of hominin male dyads, and another is the hominin female collective dyad society specialized in the quantification or detection of the results of collective execution dyads. The latter is a societal variation introduced in this paper as a completely new concept to discriminate hominin female social organization as distinct from hominin male collective organization. According to the hypothesis, these two specializations together form the attuned collective eco-social niche, but the mis-attunement or exclusion of individual dyads from the above collective eco-social niches may have caused another societal variation—the need for a hardwired solo execution dyad with modified neural dynamics (hypothesis 9). Resource proxy is the solo-only execution dyad, executor or hominin male is the collective execution dyad, and evaluator or hominin female is the dyad evolved for result quantification of executor execution. These names used for dyads are important because they emphasize the specialized division of labor or the evolution of neurodivergence to meet the demands of the environment. These hypotheses are supported by the correlation between directional brain lateralization and social hierarchy (42). The executor (hominin male) supply curve niche has an implicit assumption of social hierarchy or execution inequality and, hence, higher directional lateralization (41, 65). The lack of social hierarchy implicit in the demand curve social niche for the evaluator dyad corresponds to more bilateral brain lateralization (42) or more inter-hemisphere communication and connection. This enhances characteristics such as drawing conclusions, intuition, and analysis (48), but not particularly attuned to a hierarchy-based social system (71). ASD or resource proxy dyad has atypical lateralization (53), which affects their social communication and language. The female’s enhanced capacity for drawing conclusions (48) is of special significance, as it correlates directly to the evaluator dyad’s capability of male execution (action control) evaluation explained later and is key in supporting the proposed demand curve eco-social niche. Having defined the various external demands and specialized traits needed to meet those environmental demands, we need to acknowledge that these gender sensorimotor and cognitive differences exist (72–74), as evidenced by existing research on gender cognition.

### 7.1 Group 2: Eco-social niche hypotheses

#### 7.1.1 Hypothesis 6: Hominin male eco-social niche

Hominin males or executor dyads evolved through the co-evolution of Nash equilibrium (29, 30, 75) or co-opetition eco-social niche by recruiting a group of executor dyads with a sensorimotor balance of both superior action control and optimum action perception (hypotheses 3 and 4) to form a supply curve (29) of these hominin males in collective execution.

#### 7.1.2 Hypothesis 7: Hominin female eco-social niche

Hominin females or evaluator dyads evolved through the co-evolution of the economic demand curve (29) eco-social niche by recruiting evaluator dyads with sensorimotor polarization toward superior action perception (“agnostic of object”) for enhanced evaluator of males in action (76), but at the cost of a reduction of their own action control skills (45, 46) (hypothesis 2).

### 7.2 Group 3: Attunement and mis-attunement hypotheses

#### 7.2.1 Hypothesis 8: Attunement model hypothesis

The physical display of the unequal hominin male execution balance forming a supply curve (29) through the Nash equilibrium (29, 30, 75) coopetition (hypothesis 6) intersects with the demand curve (29) of evaluator dyads (hypothesis 7) setting the cut-off or optimum execution quantification (equilibrium price-point). This allows the female to select the superior male in execution (76) through advanced action perception or evaluator skills (hypothesis 4), leading to enhanced survivability through the multi-dimensional attuned eco-social niche.

#### 7.2.2 Hypothesis 9: Mis-attunement model hypothesis for ASD

Neurodivergent or resource proxy dyad is the breakdown or mis-attunement of the eco-social niche of coopetition interplay (hypothesis 8) between a group of individuals, as some of the individuals lack both optimal action perception (hypothesis 4) and action control capability (hypothesis 1) or the theory of mind (77). This results in the inability of the group to achieve or sustain Nash equilibrium and therefore the eco-social niche collapses for them to a solo specialization of extreme object fixation (“one of the static objects”) or enhanced systemizing skills (hyper-systemizing theory).

#### 7.2.3 Hypothesis 10: Mis-attunement model hypothesis for psychosis

Individuals with extreme evaluator (Hypothesis 2) have the neurodivergent-psychotic trait or extreme action perception leading to imaginary perception or seeing things that may not be real (diametric mind theory).

## 8 Toward interpersonal psychiatry for the autism feature space

### 8.1 ASD abilities and limitations arising from differences in cognitive information processing

In academic literature, we find clinical or medical references for ASD symptoms (78) (as outlined in Section 2) or features, but to understand the neural dynamics or information processing capabilities, we need an intermediate (79) or logical representation as identified by the embodied cognition schematic (**Figure 3**) using cognitive psychology concepts. Individuals with ASD have superior systemizing abilities (15), which in the proposed embodied cognition hypothesis (hypothesis 1) lingo means “one of the static objects”, so the internal motion in cognitive psychology terms is evolutionarily slowed down to focus more on the objects as a solo individual for enhanced innovations rather than transfer of information through social interaction to optimize the collective performance of a group. Although this ASD ability is superior for innovation (80), it does result in a spectrum of executive dysfunction (81), as it compromises the coupling of the internal and external motions impacting sensorimotor skills of action perception and control (hypothesis 1) and also active inference predictive hypothesis to control environmental (82)—clumsiness (52). Human language has developed as hominins evolved by recruiting the same neural circuits as social interactions (83), so a reduction in social interaction caused by an affinity toward objects in an atypical manner (50) also led to an atypical evolution of language skills in individuals with ASD. As we uncover the language (24) of the internal states per the Group 1 hypothesis and through the testing techniques outlined in Section 8.2, we will also be able to outline techniques to help ASD with cognitive adaptation.

### 8.2 Approach to testing embodied cognition hypotheses

**Figure 6:**
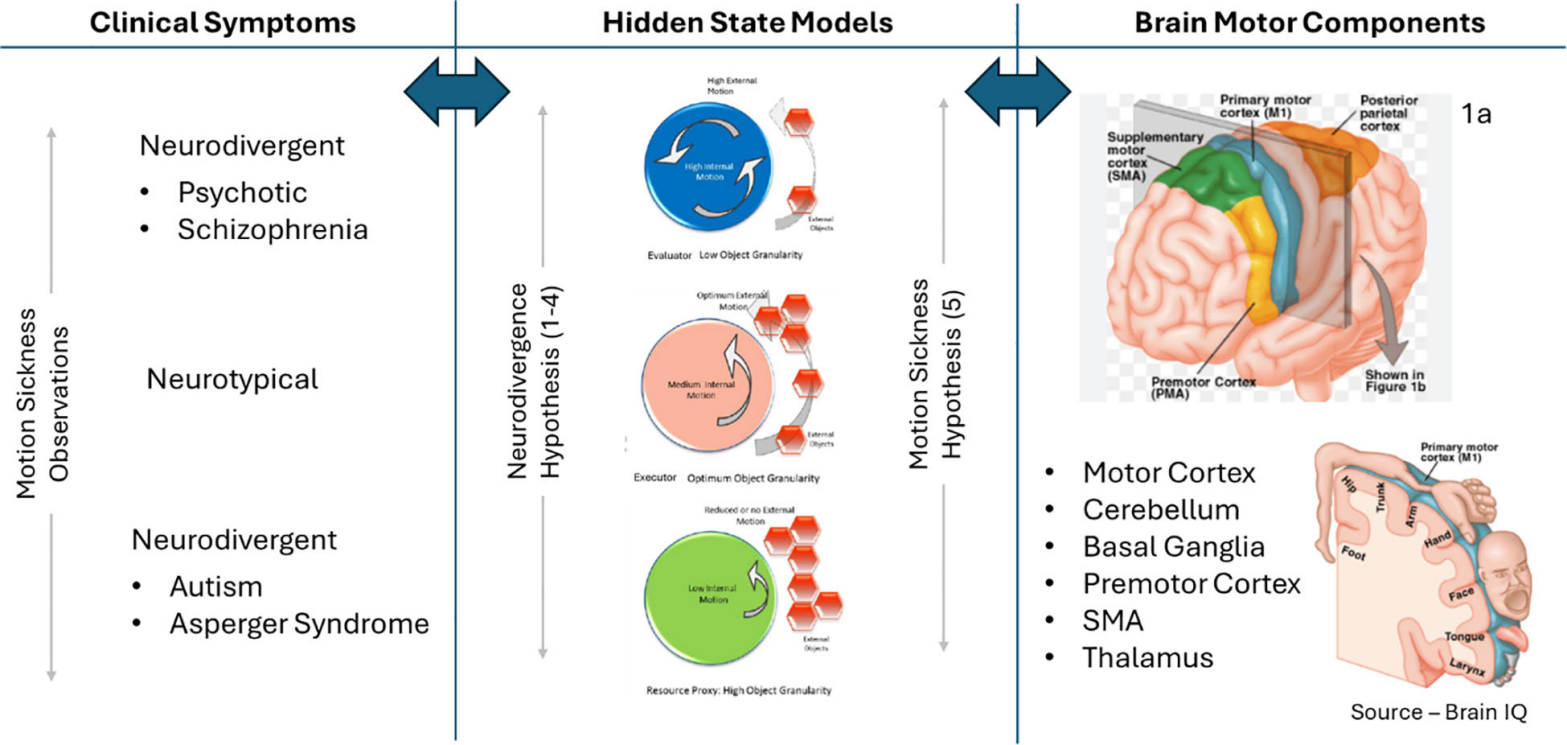
Approach to testing the hidden state hypotheses.

Motion sickness is proposed (Hypothesis 5) to be a symptom of the variability or differential in the hidden states as described in **Figure 3**, thus offering a clue into the workings of the “internal mind” or the hidden state and can be used to test the proposed embodied cognition hypotheses (hypotheses 1 to 4). We know that females generally have a higher susceptibility (3) to motion sickness, but more testing is needed in people on the autism spectrum to validate that motion sickness has a higher threshold owing to a lower internal motion state (hypothesis 1), so a greater level of external movement is needed for the additive impact to trigger motion sickness. If this test validates the proposed cognitive internal motion model in **Figure 3**, we would need to perform additional research and testing by working backward toward the predictable variations in the structure, size, and performance of the brain’s motor components—motor cortex, cerebellum, basal ganglia, thalamus, and supplementary motor area (SMA) (84)—especially the variability of the p-cell function in the cerebellum (24). Further mapping of the test subjects to the corresponding position on the hidden state spectrum as shown in **Figure 6** based on the respective neurotypical or neurodivergent clinical characteristics as well as motion sickness susceptibility will help confirm the embodied motion hypotheses if a continuum is established.

### 8.3 Approach to testing eco-social niches as well as attunement and mis-attunement hypotheses

According to the eco-social hypotheses, the economic supply and demand curve (29) forms the multi-scale dimensional eco-social niche (2) established through iterative fine-tuning and specialization of the sensorimotor skills required to establish equilibrium with the environment (27). A formal model is presented in which the influence of the market mechanism on selection is made explicit and the biological markets mirror the economic market, so the economic and social ecological models can be applied in the AI and machine learning constructs.

We restrict ourselves to biological markets in which: (1) Individuals (hominin males) do not compete over access to partners in an agonistic manner, but rather by outcompeting each other in those aspects that are preferred by the choosing party. (2) The commodity the partner has to offer cannot be obtained by the use of force but requires the consent of the partner. These two restrictions ensure a dominant role for partner choice in the formation of partnerships. In a biological market model the decision to cooperate is based on the comparison between the offers of several potential partners (hominin males), rather than on the behavior of a single potential partner, as is implicitly assumed in currently accepted models of cooperation. In our example the members of one class A (hominin females) offer a commodity of fixed value in exchange for a commodity of variable value supplied by the other class, B (hominin males). We show that when the B-class outnumbers the A-class sufficiently and the cost for the A-class to sample the offers of the B-class are low, the choosiness of the A-class will lead to selection for the supply of high value commodities by the B-class. (Ronald N0., Peter H, 1994, 1)

**Figure 7:**
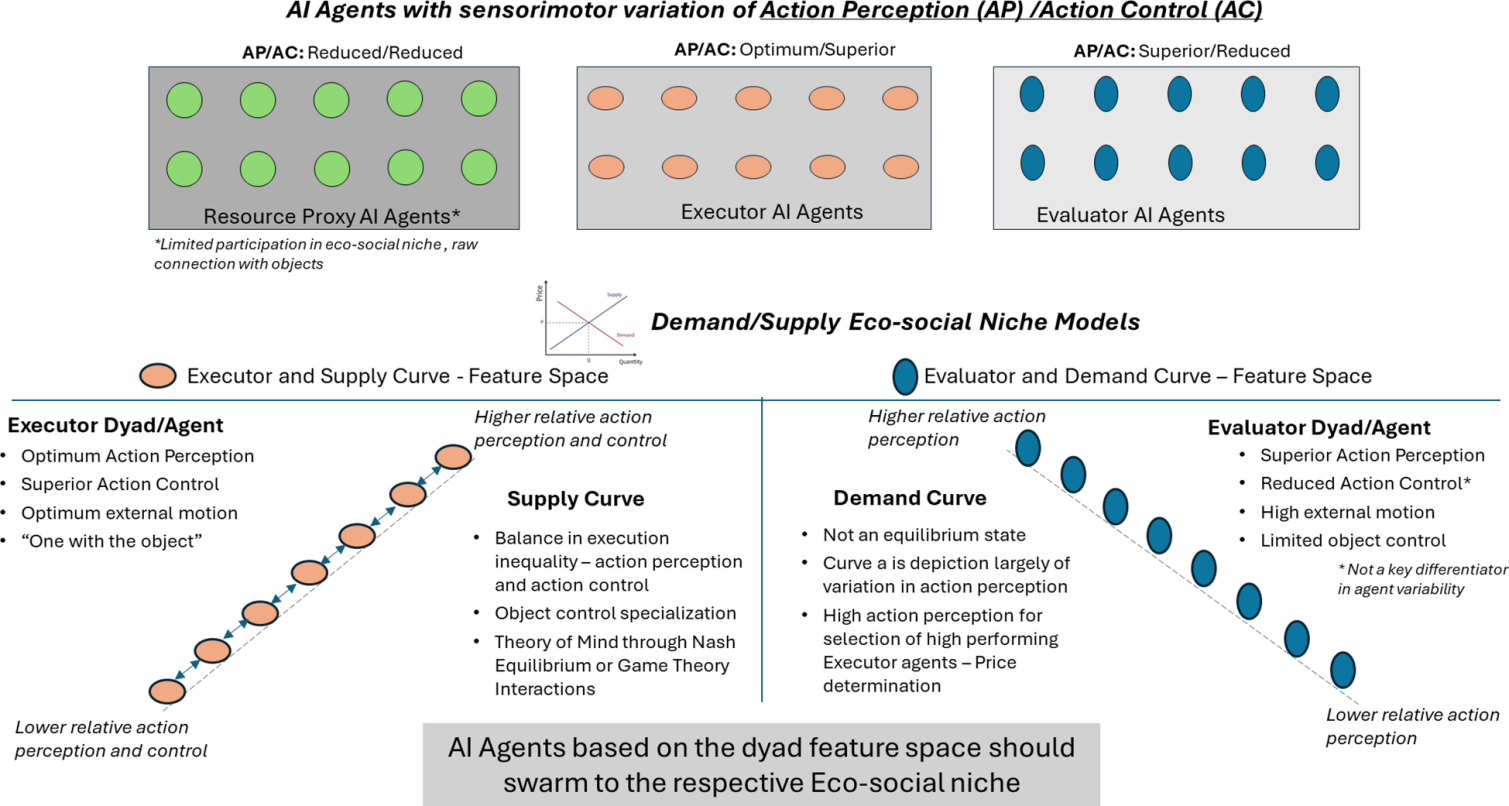
AI agent model to test the multi-dimensional hypothesis. AI, artificial intelligence.

**Figure 8:**
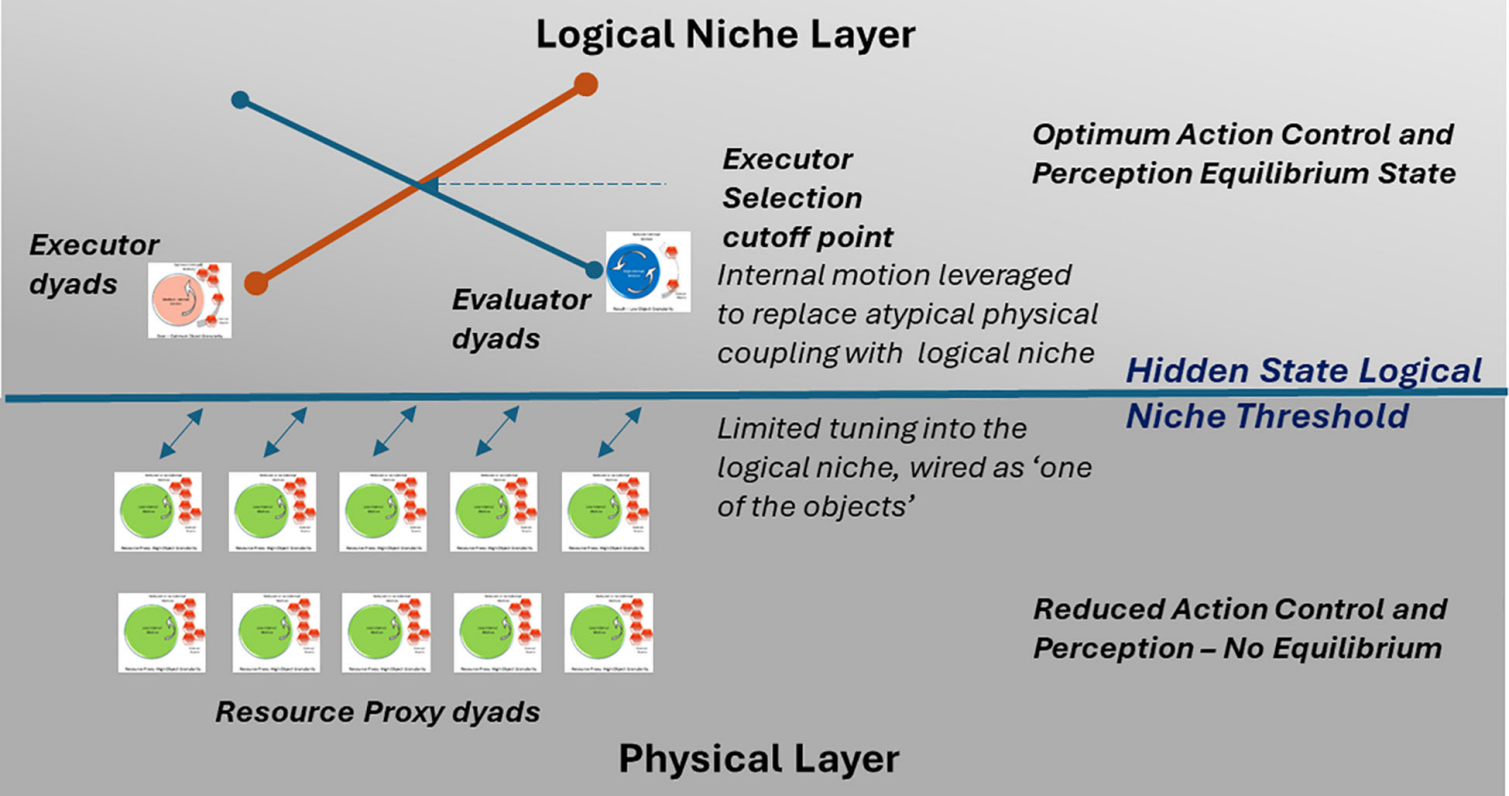
Internal motion-driven logical niche threshold between neurotypical hominins and hominins with ASD. ASD, autism spectrum disorder.

Drawing on the above approach, the 10 hypotheses defined in this paper provide discriminative information that can be modeled in a multidimensional space to demonstrate attunement as well as mis-attunement, as shown in **Figures 7** and **8**. The Nash equilibrium model of the supply curve serves as one dimension (A-class), as highlighted in hypothesis 6 as the attunement model of individual executor dyads or AI agents interacting in a balanced manner leveraging the individual sensorimotor variations for collective execution. Similarly, the female demand curve eco-social model highlighted in hypothesis 7 serves as another dimensional model based on the evaluator individual dyads (B-class) or AI agents with superior action perception skills. As per the social attunement (hypothesis 9), the agents swarming to these eco-social niche models (supply and demand curves) will result in an equilibrium state and form the social attunement model. However, AI agents with compromised action perception and action control sensorimotor skills will result in a mis-attuned state, and these agents will match the solution spaces or characteristics defined by hypotheses 1 (neurodivergent-ASD) and 10 (neurodivergent-psychotic). This would prove the validity of the eco-social niche hypotheses and the applicability of the economic principles of supply, demand, and Nash equilibrium to the hominin biological market.

Agent-based modeling in the field of ecological economics (85), socio-ecological systems (86, 87), economics, and finance (88–90) along the validation approaches (91) is gaining momentum, so the time is ripe to leverage it to push the envelope to test the model society and human health. The papers on honeybush, “Agent-based model development of complex socio-ecological systems: Methods for overcoming data and domain limitations” (92) and “An agent-based simulation model of pedestrian evacuation based on Bayesian Nash equilibrium” (93), are good examples of using agentic AI algorithms to test real-world socio-ecological and economic phenomena.

The results of the two testing approaches should lay the foundation for a holistic explanation of internal and external factors at play in the evolution and susceptibility of neurodivergence as well as a peek into the neural dynamics and component structure variations of the brain for evolving movement and cognition. I am listing a few agentic AI frameworks that have been developed and can be leveraged to test the hypotheses.

a) The enactivist^8^-inspired mathematical framework (58) for sensorimotor system (SM system), which is a special case of a transition system, can be used to model the agent environment and agent–agent interactions of attunement and mis-attunement. Sensorimotor systems can describe the body–brain system, the body–environment system, and other parts of the brain–body–environment system. The given two SM systems can be *coupled* to produce another (third) SM system (58).
b) Additionally, the proposed testing approaches can leverage the multi-agent reinforcement learning (MARL) framework (94), which outlines that interactions can be of three types: cooperative games (all agents working for the same goal), competitive games (all agents competing against each other), and mixed motive games (a mix of cooperative and competitive interactions). Studies have focused on the potential of conducting research at the intersection of some of these three axes (95). Moreover, it is worth noticing that it (96) offers a tentative glimpse of what the intersection of the three axes would look like using dynamical systems with computational simulations to address falsifiable scientific questions associated with the idea of social embodiment. This would be a positive step toward social neuro AI, debunking metaphorically the “dark matter of AI” (94).

**Figure 9:**
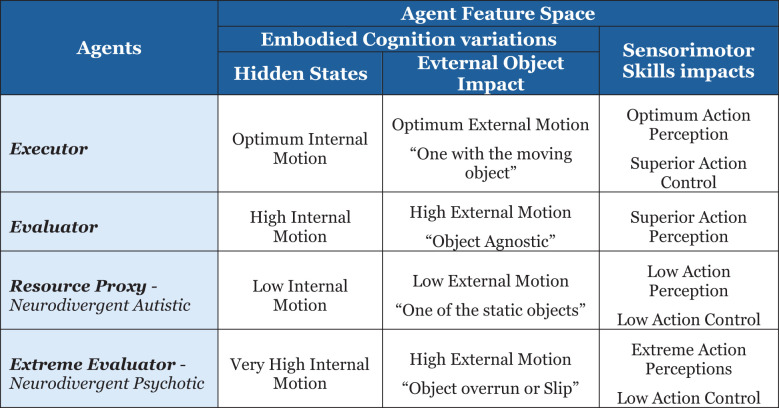
Agent’s embodied cognition feature spaces.

I will leverage the above-defined mathematical framework (58) to define the agents per the feature space defined below in **Figure 9**.

The sensorimotor skills of action perception and action control will be modeled using the hidden state motion—internal and external cognitive hypotheses. Executor with the hidden state hypothesis of optimum internal and external motion, such that the external object control allows the agent to be “one with the moving object”, leading to optimum action perception and superior action control. Additionally, define the evaluator with a hidden state of high internal and external motions, such that there is limited object control but superior action perception. Resource proxy with a hidden state hypothesis of low internal and external motions, such that the agent is “one of the static objects”, leading to low action perception and action control.

Additionally, we will define the objective function for the maximization of the survival of the group of agents as a whole in spite of varying sensorimotor skills through the MARL framework. This framework outlines that interactions can be of three types: cooperative games (all agents working for the same goal), competitive games (all agents competing against each other), and mixed motive games (a mix of cooperative and competitive interactions). Based on the varying embodied cognition dynamics, which imparts varying sensorimotor skills to be successful at one of the games, we will be able to test the eco-social niche hypothesis—do executor agents with hominin male characteristics form a supply chain curve? Do evaluator agents with hominin female characteristics form a demand curve? How do the resource proxy agents fit in to maximize overall survivability? These agent-based tests will need to be combined with the actual human-based laboratory tests (97) to evaluate how the hidden state hypothesis states—executor, evaluator, and resource proxy correlate to the clinical neurotypical, neurodivergent, and gender variations in movement, motion sickness, and corresponding brain structure (brain–body connection) to conclusively confirm the embodied cognition and eco-social niche hypotheses. There are other approaches also available in the literature that can be leveraged to fine-tune the testing strategy.

- The multi-space dimensional machine learning approach is highlighted in the paper “Interpersonal attunement in social interactions: from collective psychophysiology to inter-personal psychiatry and beyond” (2).
- Holistic approach to modeling human behavior across cognition, physiological (physical movement and resource usage), social (cooperation and competition), and economic (market dynamics) as detailed in the paper “Human behavior simulation: objectives, methodologies, and open problems” (98).

## 9 Conclusion

From a cognitive psychology perspective, my hypotheses suggest that social mis-attunement in autism stems from differences in information processing. Humans evolved using the “dialects of internalization and externalization”, i.e., through an interaction with the environment leading to an iterative enhancement as well as differentiation of the internal neural dynamics to match the evolutionary demands by impacting the objects in discriminating ways leading to the evolution of three differentiated dyads—resource proxy, executor, and evaluator. These dyads have different sensorimotor skills of action perception and action control to enable beneficial differentiation among dyads. However, these discriminating skills also result in whether these dyads are most suited to execute in isolation (resource proxy) or collectively, thus forming attunement niches, such as a supply curve niche optimized through Nash equilibrium (executor) or a demand curve eco-social niche (evaluator)—dialects of the individual and the collective. Deficits in action perception and control, as seen in the resource proxy dyad, align with cognitive impairments like the theory of mind and executive function, impacting social interactions. Understanding these mechanisms can guide future interventions to support individuals with autism in navigating social environments. I propose that in the drive to devise a multi-scale dimensional machine learning model, the authors of the paper (2) should evaluate the above-mentioned hypotheses and consider inclusion in the modeling, which will result in a positive move toward interpersonal psychiatry. This research needs to be combined with additional research to validate the hidden state hypotheses using motion sickness as a clue or peek into yet-to-be-discovered internal models of the mind and further validate the association with psychiatric illness. There is a proliferation of research and academic theories in embodied AI, which will lead the next wave of AI applications, post the text-based and agentic AI. Agent-based AI/ML models are already in use to model multiple social, ecological, and economic problems, with widespread interest among the academic community to model human behavior and the social evolution of individual differences (99). A combination of innovative hypotheses, agentic AI economic and social models, application of active Inference, embodied AI, and cutting-edge medical research (neuroscience, genetic, and ASD clinical practice) gives us a unique opportunity to solve long-standing medical and scientific problems with significant implications for autism research. We must leverage both human-based testing approaches (54, 97) and agent-based approaches (58, 94–96) to uncover the autism riddle.

## Data Availability

The raw data supporting the conclusions of this article will be made available by the authors, without undue reservation.

## Footnotes

2 Please refer to the glossary at the end of the document for detailed definition of neural dynamics, active inference, information processing, and generative.

3 Please refer to the glossary at the end of the document for detailed definition of executive function.

4 Please refer to the glossary at the end of the document for detailed definition of machine learning.

5 Please refer to the glossary at the end of the document for detailed definition of psychophysiological.

6 Please refer to the glossary at the end of the document for detailed definition of gear belt pulley and proprioception.

7 Please refer to the glossary at the end of the document for detailed definition of variational free energy.

8 Please refer to the glossary at the end of the document for detailed definition of enactivism.

## Glossary

**Table:**

**Action control**	refers to the processes or sensorimotor skills involved in regulating and guiding our actions and behaviors in response to internal goals and external stimuli. It encompasses various cognitive and motor functions that enable individuals to plan, execute, and adjust their actions effectively; leveraged in defining the embodied cognition hypotheses, especially related to hominin male neural dynamics.
**Action perception**	refers to the ability or sensorimotor skill to understand and interpret the actions of others through visual or sensory cues. This process involves recognizing movements, gestures, and intentions, allowing individuals to predict what someone may do next; leveraged in defining the embodied cognition hypotheses, especially related to hominin female neural dynamics.
**Active inference**	A normative framework that elucidates the neural and cognitive processes underlying sentient behavior, beginning with first principles. This framework posits that perception and action work in concert to minimize a shared functional known as variational free energy.
**Cognitive granularity**	is variation in object control across the three dyads—Evaluator, executor, and resource proxy—as defined by the variation in the coupling of internal movement (hidden state) and external movement. This coupling can be measured also by the sensorimotor skills of action control and action perception, as defined in the embodied cognition hypotheses.
**Cognitive psychology**	is concerned with the internal mental processes that begin with the appearance of an external stimulus and result in a behavioral response. Cognitive psychology is concerned with the mapping between both internal and external information and observed behavior. More recently, cognitive psychology has been influenced by approaches to information processing and information theory developed in computer science and artificial intelligence.
**Demand curve**	The demand curve is a graphical representation that illustrates the relationship between the price of a good or service and the quantity demanded by consumers at various price levels. The market sets the price where there is a balance between the demand and supply. In this paper, the demand curve is used as one of the eco-social niches to model hominin female mating behavior.
**Dyad**	refers to two individuals who are interconnected or interact with each other. The term is often used in various fields, such as sociology, psychology, and mathematics. Essentially, it emphasizes the significance of the interaction and relationship between the two components.
**Eco-social niche**	refers to the specific environmental and social context in which an individual or group exists, encompassing both ecological factors (such as habitat, resources, and climate) and social dynamics (including culture, community interactions, and economic conditions).
**Embodied cognition**	is a theoretical framework that posits that our cognitive processes are deeply rooted in our physical experiences and interactions with the environment. This perspective emphasizes that the mind is not separate from the body; instead, bodily states, actions, and sensory experiences play a fundamental role in shaping thought, perception, and behavior. The paper leverages this to build the embodied cognition hypotheses, which correlate external object movement to internal states of the mind.
**Enactivism**	a perspective in cognitive science and philosophy of mind, emphasizes that cognition arises from the dynamic interaction between an organism and its environment, rather than being solely a product of internal mental processes.
**Evaluator dyad**	Hominin female that evolved with demand curve features, thus having keen action perception skills to hone into the execution skills demonstrated by the executor or hominin male on the supply curve. The term “evaluator” exemplifies the keen action perception or “choosing” skills that hominin female demonstrates for natural selection of the fittest hominin males.
**Executive function**	refers to a set of cognitive processes that are essential for regulating thoughts, behaviors, and emotions in order to achieve goals. These functions are crucial for planning, decision-making, problem-solving, and self-control.
**Executor dyad**	Hominin male that executes in a collective social niche modeled after the supply curve in a co-opetition (competition and cooperation) mode, while efficiently leveraging the objects in the environment to ensure outcomes greater than the constituent parts. The term “executor” exemplifies the action control that the male dyad needs to demonstrate for it to be “chosen” by the hominin female dyad as proof of survival fitness.
**Externalization**	Active interaction with the world including other people, modifying the environment to meet their expectations through the process of externalization (active inference ^16^).
**Gear belt pulley**	is a mechanical component used in machinery to transfer power and motion between different parts of a system. It typically consists of a pulley (a wheel with a groove for a belt) and gears, which help to adjust the speed and torque of the driven components. The reference to the gear belt pulley is just used to demonstrate the close coupling of the mind and the environment, as the two ends of the pulley move in lockstep.
**Generative model**	A statistical model designed to explain the generation of observable content from unobservable, hidden (latent) causes. For instance, it clarifies the process by which a visual object gives rise to an image on the retina. Generative models serve a dual purpose: they allow the generation of novel, synthetic content and support the inference of hidden causes from observable data. From a technical standpoint, generative models encode the joint probability distribution governing both observable and hidden causes. This paper proposes the generative hypotheses of the hidden state based on the observable movement or motion phase shift between the neurotypical and neurodiverse dyads.
**Hidden states**	In a neural context, hidden states refer to internal representations or configurations of neural activity that are not directly observable but influence behavior and cognitive outcomes. This paper hypothesizes various hidden states with motion or movement as the unifying factor between internal brain state and external environmental or object impact.
**Hominin**	is a primate of a taxonomic tribe (Hominini), which comprises those species regarded as human, directly ancestral to humans, or very closely related to humans.
**Information processing**	in cognitive psychology is the flow of information through the human nervous system, involving the operation of perceptual systems, memory stores, decision processes, and response mechanisms. Information processing psychology is the approach that concentrates on understanding these operations.
**Internalization**	Vygotskian (100) take of the internal reconstruction of an external operation, further operationalized within a predictive framework.
**Machine learning**	is a subset of artificial intelligence (AI) that focuses on the development of algorithms and statistical models that enable computers to learn from and make predictions or decisions based on data.
**Nash equilibrium**	is a concept in game theory that represents a situation in which each player’s strategy is optimal, given the strategies of all other players. In a Nash equilibrium, no player has anything to gain by changing their strategy unilaterally, meaning that each player’s choice is in balance with the choices of others. The paper uses Nash equilibrium to model the “theory of mind” as an essential characteristic of neurotypical individuals but is compromised in the ASD spectrum.
**Neural dynamics**	refers to the study of how neural activity changes over time, encompassing the patterns of electrical and chemical signals in the brain. It explores how neurons interact, how these interactions give rise to various brain states, and how they contribute to cognitive processes, behavior, and sensory experiences.
**Proprioception**	is the sense of self-movement, force, and body position. Proprioception is mediated by proprioceptors, a type of sensory receptor, located within muscles, tendons, and joints.
**Psychophysiological states**	refer to the interrelated states of psychological and physiological processes in the body. These states encompass how mental processes, such as emotions and thoughts, influence physiological responses, and vice versa
**Resource proxy dyad**	is a theoretical term coined for this document to draw a correlation to the atypical object cognition characteristics of ASD, as required to explain the hypotheses later in the document.
**Sensorimotor skills**	refer to the abilities that involve coordination between sensory input and motor output—action perception and action control. These skills are crucial for performing tasks that require both sensory perception (like seeing, hearing, or touching) and physical movement.
**Supply curve**	Upward sloping curve represents the variable cost price of companies operating at their optimum efficiency to maximize their capacity while stacked up against each other to meet the market demand. Implicit in the supply curve is the intrinsic inequality or operational hierarchy of the competing companies, which form an equilibrium to meet the market demand. In this paper, the supply curve is used as one of the eco-social niches to model hominin male mating behavior.
**Variational free energy**	This is the functional (function of a function) that is minimized within the framework of active inference. It is also widely utilized in probabilistic modeling, statistical inference, and machine learning.
